# *KIBRA*, *MTNR1B*, and *FKBP5* genotypes are associated with decreased odds of incident delirium in elderly post-surgical patients

**DOI:** 10.1038/s41598-021-04416-z

**Published:** 2022-01-11

**Authors:** Mark Terrelonge, Sara C. LaHue, Christopher Tang, Irina Movsesyan, Clive R. Pullinger, Dena B. Dubal, Jacqueline Leung, Vanja C. Douglas

**Affiliations:** 1 Department of Neurology, School of Medicine, University of California, San Francisco, CA USA; 2 Department of Neurology, Weill Institute for Neurosciences, University of California, San Francisco, CA USA; 3 Department of Anesthesia and Perioperative Care, University of California, San Francisco, CA USA; 4 Cardiovascular Research Institute, University of California, San Francisco, CA USA

**Keywords:** Genetics, Medical research, Neurology, Risk factors

## Abstract

Despite the association between cognitive impairment and delirium, little is known about whether genetic differences that confer cognitive resilience also confer resistance to delirium. To investigate whether older adults without postoperative delirium, compared with those with postoperative delirium, are more likely to have specific single nucleotide polymorphisms (SNPs) in the *FKBP5, KIBRA*, *KLOTHO, MTNR1B*, and *SIRT1* genes known to be associated with cognition or delirium. This prospective nested matched exploratory case–control study included 94 older adults who underwent orthopedic surgery and screened for postoperative delirium. Forty-seven subjects had incident delirium, and 47 age-matched controls were not delirious. The primary study outcome was genotype frequency for the five SNPs. Compared with participants with delirium, those without delirium had higher adjusted odds of *KIBRA* SNP rs17070145 CT/TT [vs. CC; adjusted odds ratio (aOR) 2.80, 95% confidence interval (CI) 1.03, 7.54; *p* = 0.04] and *MTNR1B* SNP rs10830963 CG/GG (vs. CC; aOR 4.14, 95% CI 1.36, 12.59; *p* = 0.01). *FKBP5* SNP rs1360780 CT/TT (vs. CC) demonstrated borderline increased adjusted odds of not developing delirium (aOR 2.51, 95% CI 1.00, 7.34; *p* = 0.05). Our results highlight the relevance of *KIBRA, MTNR1B,* and *FKBP5* in understanding the complex relationship between delirium, cognition, and sleep, which warrant further study in larger, more diverse populations.

## Introduction

Delirium is a prevalent acute disturbance in mental status affecting more than 2.6 million hospitalized adults in the United States annually^1^. Delirium is a common complication after elective surgery in older adults, and is associated with a plethora of poor outcomes, including prolonged hospital stay, readmission, loss of independence, new or accelerated cognitive impairment, and death^2–5^. There are also numerous risk factors associated with the development of delirium. Some of the most significant risk factors for delirium are advanced age, baseline cognitive impairment, and exposure to hospital-associated stressors (e.g., surgery, anesthesia, and sleep disruption)^1^. While the field of delirium research has made remarkable progress in identifying and mitigating risk factors, less is known about endogenous, adaptive mechanisms that may prevent individuals from developing delirium despite being at risk.

The ability to maintain a baseline level of health or function through intrinsic adaptation despite exposure to stressors is a hallmark of resilience^6^. There is a growing literature on the role of resiliency in cognition, but this conceptual paradigm is rarely applied to the study of delirium. One model for resiliency is the concept of cognitive reserve, or the ability to maintain baseline cognitive function during times of physiologic stress that may have otherwise resulted in neuropathological harm^7^. Just as cognitive impairment is a risk factor for delirium, cognitive reserve may be protective. Indeed, in the Successful Aging after Elective Surgery (SAGES) study, higher baseline performance on neurocognitive testing as an indicator of cognitive reserve was protective against postoperative delirium^7^. Examining why patients at risk for delirium *do not* develop delirium offers a critical window into how vital functions are maintained in the face of common biological stressors, such as surgery.

An essential focus of inquiry is the study of genetic factors that may provide resilience to disease. As the population continues to age, there is growing interest into genetic factors that are associated with cognitive health, which may also contribute to a reduced risk of delirium. In both the general medical and postoperative setting, there is evidence to support that Apolipoprotein E e4 (*APOE4*) carrier status—the most significant known genetic risk factor for sporadic Alzheimer’s disease (AD)—is associated with postoperative delirium risk^8,9^. Conversely, less is known about whether genetic variants that are associated with cognitive resilience reduce the risk of developing delirium.

Based on the current literature, we focused on specific single nucleotide polymorphisms (SNPs) of four candidate genes associated with reduced rates or acceleration of cognitive impairment but never studied in delirium: (1) KIdney and BRAin expressed protein (*KIBRA*; rs17070145; C/T), (2) *KLOTHO* (rs9536314; T/G), (3) Sirtuin 1 (*SIRT1*; rs7896005; A/G), and (4) FK506 binding protein 51 (*FKBP5*; rs1360780; C/T). We also investigated the Melatonin Receptor-1B (*MTNR1B*) SNP rs10830963 (C/G), which was recently demonstrated in a single cardiac surgery population to be associated with reduced odds of developing delirium^10^. We sought to understand whether the absence of delirium was independently associated with increased odds of having genotypes that are associated with resiliency to cognitive impairment.

## Methods

### Participants and eligibility criteria

This prospective nested matched exploratory case–control study was derived from an initial prospective cohort investigation that took place from 2002 to 2010^8^. Individuals were eligible in the initial study if they were at least 65 years old, English-speaking, and scheduled to undergo major elective orthopedic surgery requiring anesthesia with an expected hospital length of stay of more than 48 h. From this cohort, we selected participants who self-identified as white to minimize the potential for confounding since SNP allele frequencies may vary greatly between geographic or ethnic groups^11,12^. Each study participant provided informed consent preoperatively. This study was approved by the institutional review board for human research at the University of California, San Francisco, California (IRB 10-04658 and 10-02710), was performed in accordance with their guidelines and regulations, and required informed consent from all participants.

### Participant characteristics

Participant clinical characteristics were measured during preoperative or postoperative interviews, or were abstracted from the electronic medical record. Demographics included age, race/ethnicity, and highest educational level achieved. Clinical variables collected included surgery type, the American Society of Anesthesiologists (ASA) physical status, anesthesia type (general, regional, or combined), presence of depression and functional status. Depression was ascertained using the Geriatric Depression Scale, where depression was defined by the presence of six or more symptoms of depression^13^. Functional status was ascertained using the Activities of Daily Living^14^ and Instrumental Activities of Daily Living^15^ scales.

### Neurocognitive and delirium assessments

Each participant underwent a preoperative neurocognitive evaluation approximately one week prior to their elective admission for surgery using the Telephone Interview of Cognitive Status (TICS) instrument^16^. Participant medical history, pain scale, and functional status were also assessed. The preoperative interview which was conducted prior to hospital admission included delirium screening using the Confusion Assessment Method (CAM)^17^. Patients were excluded if they were delirious on pre-operative assessment. All neurocognitive and delirium testing were performed by trained research assistants.

Postoperatively, participants were screened in person for delirium on postoperative days one and two by trained research assistants. Postoperative delirium was defined as the participant meeting CAM criteria for delirium on either the first or second postoperative day assessments. The diagnosis of delirium was confirmed by a second investigator by reviewing the information gathered by the research assistants including a written description of the patient’s mental status examination and the CAM. All evaluators of delirium were blinded to the results of the SNP analysis.

### SNP analysis

Sanger sequencing-based SNP genotyping was completed by Quintara Biosciences and included the following SNPs: *FKBP5* SNP rs1360780, *KIBRA* SNP rs17070145, *KLOTHO* SNP rs9536314, *MTNR1B* SNP rs10830963, and *SIRT-1* SNP rs7896005. These SNPs were selected based on a review of published evidence of SNPs associated with cognition, and in the case of *MTNR1B*, delirium.

### Statistical analysis

Subjects diagnosed with postoperative delirium were matched with subjects without delirium based on age (+ /− 2 years), sex, and dichotomized preoperative TICS score (cutoff score ≥ 31)^16^. Additional baseline characteristics including education, body mass index (BMI), ASA classification, anesthesia duration, length of stay, and medical history including history of dementia, depression, central nervous system (CNS) illness (including stroke, neurodegenerative disease, seizure disorder, or psychiatric disorder, among others), hypertension, congestive heart failure, and renal disease were also obtained.

The primary study outcomes were the odds of having specific genotypes in delirious patients and non-delirious controls. Baseline patient characteristics between those with and without incident delirium were compared using a Wilcoxon Signed-Ranks Test for continuous variables, given continuous variables were not normally distributed, and a Chi-Squared test for categorical variables. Conditional logistic models stratified on each matched pair were fit and evaluated using the Cochran–Mantel–Haenszel statistic to determine the association between incident delirium and genotype. Models were adjusted for education level and anesthesia duration based on a preselected *p* = 0.20 cutoff for differences in baseline characteristics. Significance for all tests was defined at the α = 0.05 level. All statistical analyses were performed using *SAS* version 9.4 (SAS Institute, Cary NC).

## Results

A total of 94 subjects were included in the study, 47 with incident delirium and 47 without. Study subjects did not show any statistically significant differences in matched baseline characteristics, history of comorbid diseases, first language, or features related to their surgery (anesthesia duration, ASA classification) (Table 1).

**Table 1:** Participant demographics and clinical characteristics in subjects with and without incident delirium.

Demographic	Delirium present N = 47	Delirium absent N = 47	*P*-value
Median age (years) (IQR)	71 (67,76)	70 (68,75)	0.97
Female sex	55.32%	55.32%	1.0
> 12 years of education	74.47%	89.36%	0.07
Medical history			
CNS illness	61.70%	55.32%	0.53
Dementia	2.13%	2.13%	1.0
Depression	42.55%	36.17%	0.53
Hypertension	65.96%	57.45%	0.32
Congestive heart failure	10.64%	4.26%	0.27
Renal disease	2.13%	4.26%	0.57
Pre-operative TICS score (≥ 31)	93.62%	93.62%	1.0
Median body mass index (IQR)	27.90 (24.70, 31.80)	27.35 (24.60, 32.00)	0.55
Median ASA class (IQR)	2 (2, 3)	2 (2, 3)	0.58
Anesthesia duration (hours) (IQR)	4.63 (3.57, 6.40)	4.13 (2.91, 6.20)	0.18

*ASA* American Society of Anesthesiology, *CNS* central nervous system, *IQR* interquartile range, *TICS* telephone interview of cognitive status.

SNP distribution by delirium status is included in Supplemental Table 1. In the genotype analysis, absence of delirium was associated with three of five SNPs (Table 2). Subjects without incident delirium had higher odds of having *KIBRA* SNP rs17070145 CT/TT (vs. CC) genotype when compared to those with delirium in adjusted models (OR 2.29; 95% CI 0.94, 5.56 *p* = 0.07; adjusted OR 2.80; 95% CI 1.03, 7.54; *p* = 0.04). Subjects without incident delirium had higher odds of having *MTNR1B* SNP rs10830963 CG/GG (vs. CC) genotype when compared to those with delirium in both unadjusted and adjusted models (OR 3.40; 95% CI 1.25, 9.21; *p* = 0.02; adjusted OR 3.88; 95% CI 1.33, 11.31; *p* = 0.01). Subjects without delirium had borderline higher odds of having *FKBP5* SNP rs1360780 CT/TT (vs. CC) in adjusted models (aOR 2.51, 95% CI 1.00, 7.34; *p* = 0.05). There was no significant association found between *KLOTHO* and *SIRT-1* genotypes and incident delirium in the unadjusted or adjusted models.

**Table 2:** Unadjusted and adjusted odds ratios for single nucleotide polymorphisms in subjects without incident delirium compared to those with incident delirium.

Single nucleotide polymorphism	Unadjusted Odds Ratio (95% CI)	*P*-value	Adjusted Odds Ratio (95% CI)	*P*-value
*FKBP5* SNP rs1360780 (CT/TT vs CC)	2.28 (0.94, 5.56)	0.07	2.51 (1.00, 7.34)	0.05
*KIBRA* SNP rs17070145 (CT/TT vs CC)	2.29 (0.94, 5.56)	0.07	2.80 (1.03, 7.54)	0.04
*KLOTHO* SNP rs9536314 (TT/GG vs TG)	0.63 (0.25, 1.64)	0.35	0.59 (0.21, 1.61)	0.30
*MTNR1B* SNP rs10830963 (CG/GG vs CC)	3.40 (1.25, 9.21)	0.02	4.14 (1.36, 12.59)	0.01
*SIRT-1* SNP rs7896005 (GG vs GA/AA)	1.77 (0.78, 4.02)	0.16	1.72 (0.73, 4.05)	0.21

*CI* confidence interval, *FKBP5* FK506 binding protein 51, *KIBRA* kidney and brain expressed protein, *MTNR1B* Melatonin Receptor-1B, *SIRT 1* sirtuin 1, *SNP* single nucleotide polymorphism.

## Discussion

In this matched exploratory case–control study of 94 participants who underwent elective orthopedic surgery, of whom 47 developed incident postoperative delirium and 47 did not develop delirium, individuals without delirium had an increased adjusted odds of having the *KIBRA* rs17070145 CT/TT and *MTNR1B* rs10830963 CG/GG genotypes. While there is data supporting the role of the *KIBRA* conferring cognitive resilience in older adults, this is the first study investigating and demonstrating an association between delirium and *KIBRA*. This is also the first demonstration of an association between delirium and *MTNR1B* in a non-cardiac surgery population. Interestingly, individuals without delirium may also have increased odds of *FKBP5* SNP rs1360780 CT/TT, which is associated with worse cognition in aging and after stress. The identification of genetic differences between those who do and do not develop delirium may point to potential endogenous, adaptive mechanisms that may prevent individuals from developing delirium despite being at risk.

One of the most significant risk factors for delirium in older adults is cognitive impairment^1^. The *KIBRA* rs17070145 T allele has been demonstrated in multiple studies to be associated with a reduced risk of AD, which is potentially mediated by its inhibition of β-amyloid neuronal apoptosis^18,19^. In one study of 602 cognitively normal adults who underwent Aβ-amyloid PET imaging, and were followed longitudinally for six years, those who were *APOE4* carriers but also *KIBRA* T allele carriers experienced significantly slower rates of cognitive decline and hippocampal atrophy than those who were non-*KIBRA* T allele carriers^18^. As such, the *KIBRA* T allele may confer cognitive resilience even in a population with increased vulnerability to developing cognitive decline. That we found patients with delirium were more likely to have the *KIBRA* CC genotype provides additional compelling evidence for the link between delirium and cognitive impairment.

While cognitive function can be compromised by neurodegeneration, exogenous stress is also an established risk factor for impairment of learning and memory and so provides an important model for investigating cognitive resilience^20^. The *FKBP5* gene encodes FK506-binding protein 51 (FKBP51), and the T allele carriers tend to exhibit increased mRNA expression levels of FKBP5^21^. There is increasing interest in the interaction between the *FKBP5* genotype and environmental stressors, where induction of FKBP5 and prolonged cortisol responses are associated with several neuropsychiatric disorders, though with some conflicting results^22,23^. To our knowledge, while there are no genetic association studies between FKBP5 and AD, there is growing appreciation for the importance of FKBP51 in the aggregation of tau seen in neurodegenerative diseases such as AD^21,24^. In a transgenic rodent model of AD, FKBP51 overexpression was associated with phosphorylated tau and a reduced number of hippocampal neurons, both hallmarks of AD pathology^24^. In human brains, increased FKBP51 levels were associated with increased Braak staging, suggesting that increased FKBP51 may also contribute to AD progression, potentially due to the pathogenesis exhibited in the rodent models^24^. Based on this literature, our hypothesis was that patients with delirium would have an increased odds of carrying the T allele; that we found borderline increased odds of the *FKBP5* CT/TT genotype in adults without delirium runs counter to the expected outcome and demonstrates the need for additional larger studies in delirium-specific populations.

While cognitive impairment is an important independent risk factor for delirium, there is growing recognition of a shared risk factor for both delirium and cognitive impairment: sleep disruption^25–27^. Both delirium and sleep disruption are provoked by the use of several medications commonly used in hospitalized patients, including benzodiazepines, opiates and corticosteroids^26^. Delirium and sleep disruption may also overlap in pathophysiology, as both are associated with acetylcholine and melatonin dysregulation, and dysfunction of the prefrontal and parietal lobes as demonstrated using electroencephalography and functional neuroimaging^26,28^. One meta-analysis investigating the association between perioperative sleep disturbance and postoperative delirium demonstrated a pooled odds ratio of 5.24 (95% CI 3.61–7.60), though this was limited by the heterogeneity with which both sleep disturbance and delirium were measured. There is significant interest in improving sleep for hospitalized patients to reduce the risk of delirium, including care pathways providing non-pharmacologic means for improving sleep (e.g., ear plugs, eye masks), or the use of supplemental melatonin, which is a regulator of the circadian rhythm^29^. The *MTNR1B* gene encodes the melatonin receptor MT2, a G protein-coupled receptor that is expressed in many tissues and acts as a receptor for melatonin, a hormone released from the pineal gland that is important for regulation of circadian rhythms^10^. The *MTNR1B* G allele of the SNP rs10830963, which was found to be more common in adults without delirium in our study, is associated with altered melatonin signaling, specifically both a longer duration of elevated melatonin levels and a delayed off time^30^. Reductions in circulating melatonin, as measured in serum and urine metabolites, have been associated with delirium in hospitalized patients^28^. Just as individuals at risk for delirium are provided with supplemental melatonin, it is possible that having elevated and prolonged levels of melatonin can be helpful in a hospital environment where disrupted sleep is common. Interestingly, our finding is opposite the only known study investigating this gene in relation to delirium, specifically in a small population of subjects undergoing cardiac surgery^10^. Despite the observed association between melatonin dysregulation and delirium, trials of exogenous melatonin or the melatonin agonist ramelteon yielded mixed results^10^. It is possible that classifying the presence of *MTNR1B* genotype in participants of these trials may provide a role in understanding who may benefit from these delirium prevention therapies.

There is no medication that prevents or treats delirium at this time. Current delirium management focuses on non-pharmacological intervention, primarily focusing on frequent reorientation, mobilization, maintenance of sleep–wake cycles, avoiding constipation and urinary retention, and minimizing polypharmacy^31,32^. This multicomponent approach may reduce delirium incidence by 30–40%^31^. However, these interventions require significant organizational resources and are therefore best directed at patients with the highest risk. This also indicates that there are at least 60% of hospitalized individuals who develop delirium despite these evidence-based, intensive care pathways. There remain major shortcomings in our ability to accurately identify who is at greatest risk of developing delirium, and medical therapies for its prevention and treatment do not exist. Identifying associations between the development of postoperative delirium and specific genotypes may lead to more targeted delirium prediction models for resource allocation as well as targeted therapeutic trials stratified by genetic profiles.

This study features several limitations. This study is restricted to previously collected specimens. For this reason, it is possible that there is confounding by factors from the initial study that we are not accounting for, including any ascertainment bias there that may have fed into our nested study. Further, the sample size is limited by specimen availability and so it is possible that we lacked sufficient specimens to detect differences between delirious and non-delirious participants for the other SNPs. Participants were only screened for delirium on postoperative day one and two, and so it is possible that a small number of delirious cases were missed, particularly cases that occurred several days after surgery. Dementia was determined through review of the medical records and so it is possible that preoperative dementia was underreported. However, since the prevalence of dementia was very low in this cohort this is unlikely to affect our results. In addition, preoperative mild cognitive impairment was not measured in this study, and it is possible that differences in the prevalence of mild cognitive impairment among groups biased the results; future studies should measure this variable preoperatively. While we limited to this study to a single race to help minimize the effect of different allelic frequencies in different races, this limited the number of specimens available. This is in addition to fluency in English being an inclusion criterion for the original study, further limiting recruitment of a more diverse group of participants. Limiting the analysis to a single race should not prevent generalization of the results to other races, but the associations found here warrant further study in larger multi-racial populations.

## Conclusions

In a population of postoperative older adults, delirium status was associated with three SNPs: *KIBRA* SNP rs17070145, *MTNR1B* SNP rs10830963, and *FKBP5* SNP rs1360780. Those without postoperative delirium were more likely to have genotypes associated with cognitive resilience in older adults (*KIBRA* SNP rs17070145 CT/TT) and beneficial changes in melatonin signaling (*MTNR1B* SNP rs10830963 CG/GG). However, they may also have increased odds of *FKBP5* SNP rs1360780 CT/TT, which is associated with worse cognition in aging and after stress. These results highlight the complex relationship between delirium, cognition, and sleep, and warrant further study in larger, more diverse populations.

## Supplementary Information

Supplementary Information.

## Data Availability

The datasets generated and analyzed during the current study are not publicly available due to them containing information that could compromise participant privacy. The data however are available from the corresponding author (SCL) on reasonable request.
