# Supplementary material for: KIBRA, MTNR1B, and FKBP5 genotypes are associated with decreased odds of incident delirium in elderly post-surgical patients
Source: Sci Rep. 2022 Jan 11;12:556. doi: 10.1038/s41598-021-04416-z (PMC8752781; doi:10.1038/s41598-021-04416-z)
Supplement: Supplementary file 1 — Supplementary Information. [file 41598_2021_4416_MOESM1_ESM.docx]

**Supplemental Table 1: Distribution of single nucleotide polymorphisms by delirium status.**

| Single Nucleotide Polymorphism | Delirium  N=47 | No Delirium  N=47 |
| --- | --- | --- |
| *FKBP5* SNP rs1360780, No. (%) |  |  |
| C | 26 (55.32) | 17 (36.17) |
| C/T | 16 (34.04) | 26 (55.32) |
| T | 5 (10.64) | 4 (8.51) |
| *KIBRA* SNP rs17070145*,* No. (%) |  |  |
| C | 27 (57.45) | 18 (38.30) |
| C/T | 12 (25.53) | 24 (51.06) |
| T | 8 (17.02) | 5 (10.64) |
| *KLOTHO* SNP rs9536314, No. (%) |  |  |
| G | 1 (2.13) | 1 (2.13) |
| G/T | 16 (34.04) | 12 (25.53) |
| T | 30 (63.83) | 34 (72.34) |
| *MTNR1B* SNP rs10830963*,* No. (%) |  |  |
| C | 32 (68.09) | 20 (42.55 |
| C/G | 14 (29.79) | 22 (46.81) |
| G | 1 (2.13) | 5 (10.64) |
| *SIRT-1* SNP rs7896005*,* No. (%) |  |  |
| A | 6 (12.77) | 7 (14.89) |
| A/G | 18 (38.30) | 24 (51.06) |
| G | 23 (48.94) | 16 (34.04) |

Abbreviation: FKBP5:FK506 binding protein 51; KIBRA: kidney and brain expressed protein; MTNR1B: Melatonin Receptor-1B; SIRT 1: sirtuin 1; SNP: single nucleotide polymorphism
